# Improving an Electronic Health Record–Based Clinical Prediction Model Under Label Deficiency: Network-Based Generative Adversarial Semisupervised Approach

**DOI:** 10.2196/47862

**Published:** 2023-06-13

**Authors:** Runze Li, Yu Tian, Zhuyi Shen, Jin Li, Jun Li, Kefeng Ding, Jingsong Li

**Affiliations:** 1 College of Biomedical Engineering and Instrument Science Zhejiang University Hangzhou China; 2 Institute for Artificial Intelligence in Medicine, School of Artificial Intelligence Nanjing University of Information Science and Technology Nanjing China; 3 Department of Surgical Oncology Zhejiang University School of Medicine Second Affiliated Hospital Hangzhou China

**Keywords:** semisupervised learning, generative adversarial network, network analysis, label deficiency, clinical prediction, electronic health record, EHR, clinical prediction, adversarial network, data set

## Abstract

**Background:**

Observational biomedical studies facilitate a new strategy for large-scale electronic health record (EHR) utilization to support precision medicine. However, data label inaccessibility is an increasingly important issue in clinical prediction, despite the use of synthetic and semisupervised learning from data. Little research has aimed to uncover the underlying graphical structure of EHRs.

**Objective:**

A network-based generative adversarial semisupervised method is proposed. The objective is to train clinical prediction models on label-deficient EHRs to achieve comparable learning performance to supervised methods.

**Methods:**

Three public data sets and one colorectal cancer data set gathered from the Second Affiliated Hospital of Zhejiang University were selected as benchmarks. The proposed models were trained on 5% to 25% labeled data and evaluated on classification metrics against conventional semisupervised and supervised methods. The data quality, model security, and memory scalability were also evaluated.

**Results:**

The proposed method for semisupervised classification outperforms related semisupervised methods under the same setup, with the average area under the receiver operating characteristics curve (AUC) reaching 0.945, 0.673, 0.611, and 0.588 for the four data sets, respectively, followed by graph-based semisupervised learning (0.450, 0.454, 0.425, and 0.5676, respectively) and label propagation (0.475,0.344, 0.440, and 0.477, respectively). The average classification AUCs with 10% labeled data were 0.929, 0.719, 0.652, and 0.650, respectively, comparable to that of the supervised learning methods logistic regression (0.601, 0.670, 0.731, and 0.710, respectively), support vector machines (0.733, 0.720, 0.720, and 0.721, respectively), and random forests (0.982, 0.750, 0.758, and 0.740, respectively). The concerns regarding the secondary use of data and data security are alleviated by realistic data synthesis and robust privacy preservation.

**Conclusions:**

Training clinical prediction models on label-deficient EHRs is indispensable in data-driven research. The proposed method has great potential to exploit the intrinsic structure of EHRs and achieve comparable learning performance to supervised methods.

## Introduction

The recent rise of observational biomedical research, driven by greatly expanding electronic health records (EHRs) and the prevalence of machine learning methods, has drawn great attention [1-4]. Conventional strategies tend to screen out subgroups of interest based on expert supervision or established risk factors. An alternative data-driven paradigm extracts underlying subtypes by comprehensively profiling the longitudinal irregularity, interdimensional heterogeneity, and intrinsic homogeneity of the database, thus progressively facilitating the practice of precision medicine. For instance, the Electronic Medical Records and Genomics (eMERGE) network [5] leverages expertise from multiple institutions and communities to integrate biorepositories and EHRs to support genomic research. Observational research approaches exhibit both potential and challenges for more sophisticated data analysis.

However, the acquisition of realistic data, especially data labels, is still restricted when confronting concerns about system security, patient privacy, and intellectual property protection [6,7]. Excluding data and labels may be ubiquitous during the data collection phase. Long-term studies often lack sufficient time to gather data and have no control over the switching behaviors of patients [8,9], resulting in the loss of accurate outcome measurements.

Restrictions on transferring intellectual property among different institutions hinder the sharing of data, which is expected to be complete. Additionally, some expertise-requiring annotations are tedious and have no guarantee of correctness [10]. Generally, label deficiencies occur frequently when analyzing observational EHR data.

There have been some attempts to address insufficient labeling by realistic synthesized EHR (RS-EHR) generation. One approach to RS-EHRs is knowledge-based [11,12]. Such approaches combine publicly available statistics, clinical practice guidelines, and medical coding dictionaries to improve the fidelity of generated EHRs. However, the models are still restricted to development, testing, and public demonstrations.

Another strategy is data-driven. Generative adversarial networks (GANs) are a new class of methods for obtaining realistic synthesized data [13,14]. The philosophy of GANs is to train two networks, one generating fake samples and the other discriminating fake and real samples, in a min-max game until equilibrium is achieved, indicating that the generated fake samples cannot be distinguished from the real samples. There has been some work on applying state-of-the-art GANs to generate synthesized EHR data sets [15,16]. However, these studies have not fully applied the generated data to augment EHR computational phenotyping and classification. GANs for few-labeled data are still unlikely to recover the whole distribution of labels from the raw data set due to imbalanced labeling. Additionally, there are some arguments that GAN-generated samples are likely to copy real samples exactly, which is a potential violation of privacy [17,18].

Semisupervised learning (SSL) is a set of techniques that are usually adopted to leverage unlabeled data and an underlying data set structure. With a relatively small set of labeled data compared to that needed in supervised learning (SL), SSL can still display decent learning performance. Some previous studies used SSL to phenotype EHR databases [19,20]. These studies achieved excellent performance in EHR-based risk prediction, but the feature dimensions were restricted to discrete medical codes. GANs were adopted to boost the SSL [21], but as mentioned above, the generator was trained to eventually remember exact copies of the samples for the limited span of an EHR data set in a discrete and high-dimensional space, which therefore raised privacy concerns. SSL is a powerful tool for label-deficient circumstances but needs specifications for observational research.

Network analysis is a solution to both obstacles. Encoding the similarities among patients into their connections protects their identities. The input of the analysis is only the network structure and embedded vectors. Network analysis is the basis for manifold learning, which has an advantage in approximating the data structure in a high-dimensional space. Many manifold-based methods have prevailed in intuitively visualizing and phenotyping coordinated data sets [22-24]. Additionally, there have been quite a few attempts to extend deep learning to irregular data structures, such as graphs. Several studies have shown great performance in representative learning with SSL [25-28], and endeavors have been made to apply GANs to graphs [29-31].

However, few studies have considered exploiting the inherent network structure of an EHR database in SSL tasks. GANs on networks have not been fully investigated in terms of privacy preservation. Additionally, under various label-deficient situations, the performance remains to be evaluated. It is very promising to scale SSL and GANs to the graph structure extracted from an EHR database and to thereby acquire a new perspective on EHR data sets.

For this paper, the main contributions are as follows: (1) This study tries to address limitations due to existing label deficiency in observational EHR analytical research by extending the network analysis pipeline to EHRs. A boosting learning model is proposed by applying GAN-boosted SSL to network data extracted from label-deficient coordinated EHRs. (2) Experiments are conducted on 3 public data sets as well as one from the First Affiliated Hospital of Zhejiang University, and they are evaluated by prediction metrics that are compared to conventional learning methods. The proposed method shows superior performance over conventional semisupervised methods and indicates comparable performance with supervised learning methods when data are fully labeled. (3) To ensure the utility of the proposed model, further evaluations of data quality, nondisclosure, and memory space consumption are performed. The proposed method shows higher data fidelity, lower precision metrics against compromised attack, and less graphics processing unit (GPU) memory consumption over conventional semisupervised methods.

## Methods

### Data Set Structure Conversion to a Graph

Graph structure definition and semisupervised learning on graph formularization are shown in Multimedia Appendix 1A [31-37]. According to the well-accepted assumption that a manifold is locally Euclidean in topological space, it is plausible to represent a data set X with a graph G. However, this conversion rule should be scrutinized. First, it depends on the number of edges |E| that comprise the edge set. |E| should be restricted to a range that avoids disconnected components and short circuits that obscure structural information. Second, the neighborhood searching strategy should be scalable to feature value scales and effective in practice. Third, the local density variance should be preserved during conversion, which means that the weights of edges should not be binary.

To circumvent this problem, the k-nearest neighbors (k-NN) method was selected to convert the original data set into a graph measure space. As its name indicates, the k nearest points in the Euclidean space of point x are identified as its neighbors, N_k_ (x). Each edge weight w_ij_ is refined with the Jaccard coefficient:



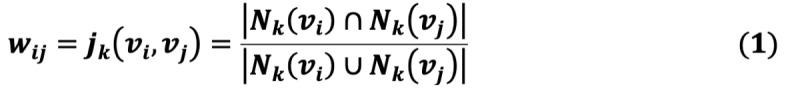



The Jaccard coefficient addresses the unified weight problem brought by k-NN searching and restricts the weights to [0,1], which scales the local densities as node degrees: deg(v_i_ ) = ∑_j∈N_k(v_i )_w_ij_. Additionally, when the lower bound is reached, the edge is removed from the graph, and eventually, nodes with degree zero will be considered noise and therefore removed. The final graph serves as one of the inputs of the GAN.

### GANs for Graphs and Their Modified Losses

In this study, we focus on generating vectorized fake samples by the use of both the graph structure and coordinated features. The coordinated features of the graph structure are acquired by feeding the Jaccard graph into large-scale information network embedding [38], explicitly setting the output dimension as half of d.

The fundamentals of GAN [32,33] are presented in Multimedia Appendix 1C [31-37]. Nevertheless, it is important to take into account the unconventional loss of semisupervised adversarial learning, as insufficient labels do not effectively minimize the current adversarial learning loss. The generator is trained to produce samples that bridge the density gap between samples from distinct classes. In the case of binary classification tasks, the 2 classes are “true” and “false.” By expanding the density gap between labeled true samples, labeled false samples, and generated density gap samples, the adjusted discriminator loss can enhance semisupervised learning performance. The refined discriminator loss *L_D_* for SSL purposes comprises semisupervised loss, entropy loss, and class distance. (1) Semisupervised loss: there are two terms; the first is the supervised loss calculated by cross-entropy between the labels and prediction. The second emphasizes the loss due to incorrect classification by SSL. λ_0_ is a hyperparameter that balances these 2 terms.


loss_semi_ = loss_sup_ + λ_0_loss_un_
= –E_(_xi∈XL_) log *P*(y_i_ | x_i_,y_i_ < M)
– λ_0_ (E_xi∈XU_ log(1 – *P*(*M* | x_i_)) + E_xi~G(z)_ log *P*(M | x*_i_*)) **(2)**

(2) Entropy regularization [39,40]: this term calculates the entropy of a distribution over all labels M to enhance the certainty of the prediction.



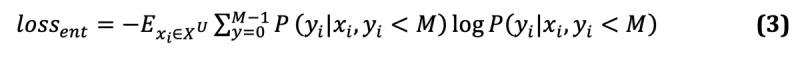



(3) Cluster distance loss [31]: this term tends to enlarge the density gap so that samples from different classes are separate. h^n^(x) is the last-layer output of the discriminator.



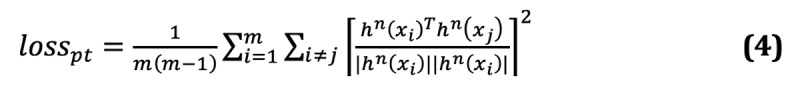



The final loss term for the discriminator generator is


loss_D_ = loss_Dwgp_ + loss_semi_ + loss_ent_ + loss_pt_ **(5)**

The loss of generator _LG_ is also modified by adding the term (4). The final loss term for the training generator is


loss_G_ = loss_Gwgp_ + loss_pt_ **(6)**

**Figure 1:**
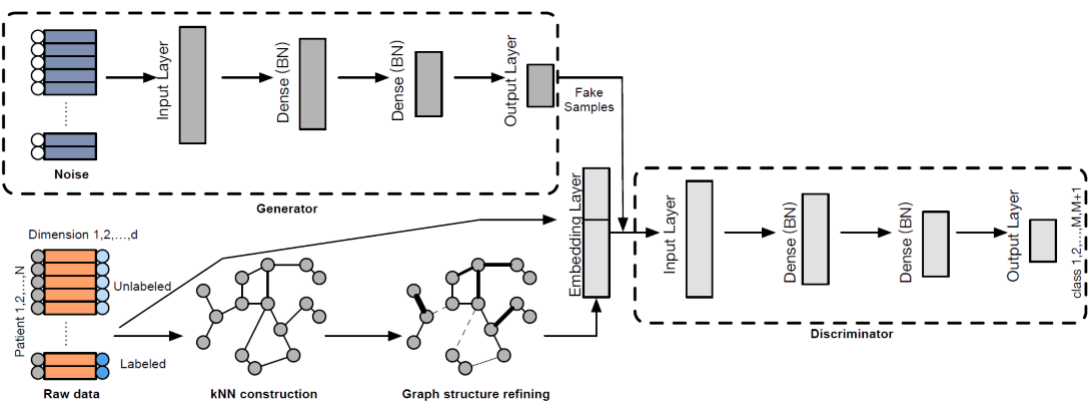
An overview of the proposed model. Real samples are extracted from the k-NN graph by the embedding layer, and the fake samples generated by the generator have the same dimension. Both real and fake samples are fed into and backpropagated from the discriminator in minibatches. The output layer of the discriminator is softmax. The embedding layer is a pretrained large-scale information network embedding [32]. BN: batch normalization; k-NN: k-nearest neighbors.

The network structure is illustrated in Figure 1. During the training phase, real embeddings and fake inputs of the same size are fed into the network separately in batches, with the goal of optimizing the aforementioned losses of the discriminator and generator. During 1 training epoch, batches of real labeled data, real unlabeled data, and fake data are fed into the network to calculate different loss terms for optimization. Batch normalization is conducted. After training, the discriminator loss is expected to be stable and could be exploited as a classifier for testing samples and predictions. The generator is suitable for measuring data quality and preserving privacy.

### Data Sets and Experimental Setup

EHR data sets were obtained from public resources, including University of California Irvine Machine Learning Repository Type 2 Diabetes 30-Day Readmission (UCI-T2D) [41]; Surveillance, Epidemiology, and End Results Ovarian Cancer (SEER-OVC) [42]; and Surveillance, Epidemiology, and End Results Colorectal Cancer (SEER-CRC) [42]. The dimensional information is summarized in Table 1. Another colorectal cancer data set from the Second Affiliated Hospital Zhejiang University School of Medicine (SAHZU-CRC) was selected to investigate feasibility in practical situations. These data sets were selected because they are long-term follow-ups, the labels of which take much time and effort to obtain and are likely to be missing due to regulations on data collection. The selected features included basic demographics, medication, clinical codes, stage codes, laboratory variables, and dispositions. A basic description of the data sets and preprocessing is provided in Multimedia Appendix 1B.

We trained the proposed models for a maximum of 200 epochs using Adam optimization with a learning rate of 0.003 and a momentum of 0.5. The batch size was 128. For each class, the rate of labeled points (the label rate is the percentage of labeled points among all points) increased progressively from 5% to 25% with a step of 5%. The number of test sets was set as 20% of the data set.

**Table 1:** Dimensional description of the selected data sets.

Data sets	Records	Categorical variables	Numerical variables	Preprocessed dimensions	Labeling standard
University of California Irvine Machine Learning Repository Type 2 Diabetes 30-Day Readmission	61,675	44	8	57	Readmission in 30 days
Surveillance, Epidemiology, and End Results Ovarian Cancer	10,038	18	3	34	Survival over 5 years
Surveillance, Epidemiology, and End Results Colorectal Cancer	40,014	7	2	14	Survival over 5 years
Second Affiliated Hospital of Zhejiang University	1244	8	2	14	Survival over 5 years

We compared the model with the following baselines: (1) supervised learning methods, including logistic regression (LR), a support vector machine (SVM), and a random forest (RF) and (2) SSL methods, including graph-based semisupervised learning (GSSL) and label propagation (LP). All these methods are run using the *scikit-learn* Python package. The graph convolutional network (GCN) [25,27], a state-of-the-art graph-based semisupervised deep learning method, is also considered a competing method. To measure the classification performance, the accuracy and recall—for the important purpose of excluding false negative cases to conserve medical resources—and the area under the receiver operating characteristics curve (AUC) were selected as metrics. Each metric represented the average of 30 repetitions of 10-fold cross-validation training.

### Ethical Considerations

This study did not involve any human or animal experiments. The UCI-T2D, SEER-OVC, and SEER-CRC data sets are public, and we complied with their ethical requirements. We also used a colorectal cancer–specific disease cohort of the Second Affiliated Hospital Zhejiang University School of Medicine; this was approved by the Human Research Ethics Committee of Zhejiang University in August 2017 (2017-067).

## Results

### SSL-based Classification of EHR Data

In the aforementioned experiments, the proposed method for semisupervised classification outperformed related semisupervised methods by a decent margin. Basic graph semisupervised methods (ie, GSSL) are limited in classification performance, mostly due to their assumption that edges encode only the similarity of nodes. The spectral methods (LP and GCNs) do not perform well, perhaps because their low-order approximation may smooth the frontiers in the graph. Neither of these 2 methods consider the local properties of the input graph, and under some circumstances, they classify the majority of nodes into 1 class. Additionally, at a 10% to 15% label rate, the proposed method achieves the best performance on SEER-OVC, SEER-CRC, and SAHZU-CRC (Table 2). The AUCs declined as label rates continued to rise. GCNs, as the state-of-the-art semisupervised deep learning method, had somewhat better results for a data set with a size that can be handled by a GPU, but still exhibited worse performance than the proposed method, presumably due to oversmoothing the graphs and having less refined loss.

**Figure 2:**
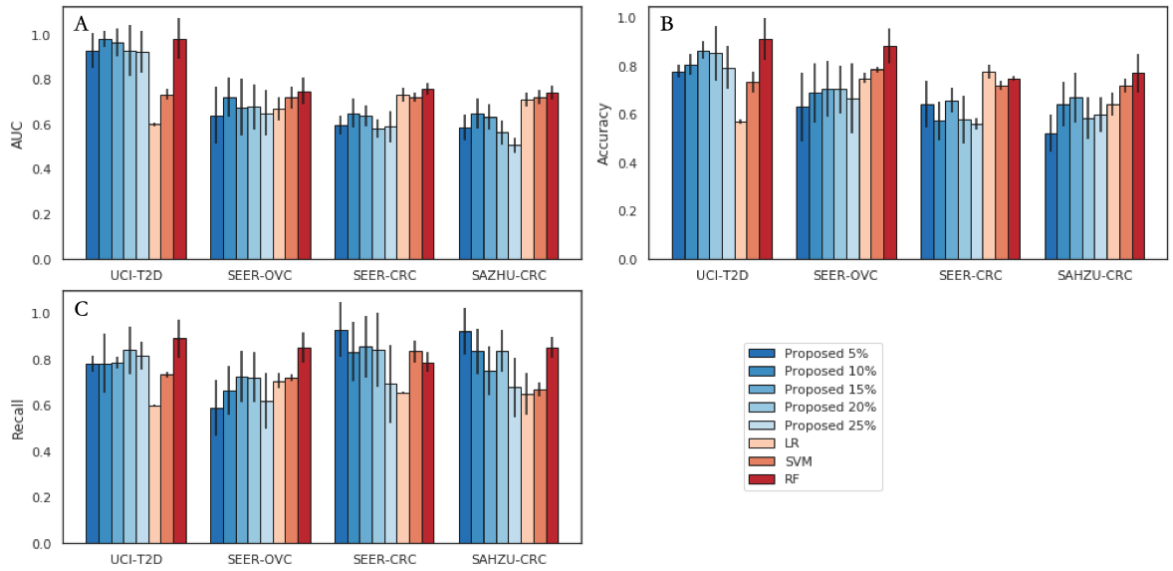
Summary of the results of the classification of the proposed method versus those of the conventional supervised learning methods. (A) AUC; (B) accuracy; (C) recall. The proposed method was evaluated under progressively increasing label rates. The supervised learning models were trained on fully labeled data. The error bars indicate the SD for each metric. AUC: area under the receiver operating characteristics curve; LR: logistic regression; RF: random forest; SAHZU-CRC: Second Affiliated Hospital of Zhejiang University Colorectal Cancer; SEER-CRC: Surveillance, Epidemiology, and End Results Colorectal Cancer; SEER-OVC: Surveillance, Epidemiology, and End Results Ovarian Cancer; SVM: support vector machine; UCI-T2D: University of California Irvine Machine Learning Repository Type 2 Diabetes 30-Day Readmission.

In regard to supervised learning, as shown by the bars in Figure 2A, even with a label rate of 10%, SSL on a graph with a GAN performed comparably to the supervised learning methods. As the portion of labeled data increased, the learning performance progressively increased, which is a consequence of the abundant information of the label distribution over the constructed graph. However, as the label rate continued to rise, the performance decreased because of mode collapse and overfitting. As the error bars show, with 10% labeled data, the standard deviations of the proposed model are slightly larger, as shown in Figures 2A and 2C, indicating a limitation of our proposed method; it only applies to certain low label-rate circumstances. When the labels are sufficient, more robust SL methods are better. However, some poorly trained and undertuned SL methods show far worse metrics in testing. Additionally, as the vector dimensions, shown in Table 1, decreased somewhat, the learning performance showed a significant decrease. This is perhaps a consequence of the lack of dimension diversity for similarity encoding and the local graph structure.

**Table 2:** Summary of the results of the classification AUCs for semisupervised learning methods under progressively increasing label rates. The learning performance of the graph convolutional network on the large data sets—that is, data sets other than Second Affiliated Hospital of Zhejiang University Colorectal Cancer—is unavailable due to memory limits.

Label Rate		5%, AUC^a^	10%, AUC	15%, AUC	20%, AUC	25%, AUC
**University of California Irvine Machine Learning Repository Type 2 Diabetes 30-Day Readmission**						
	GSSL^b^	0.450	0.472	0.523	0.542	0.602
	LP^c^	0.475	0.475	0.564	0.585	0.566
	Proposed	0.929	0.979	0.964	0.930	0.924
**Surveillance, Epidemiology, and End Results Ovarian Cancer**						
	GSSL	0.454	0.512	0.537	0.591	0.591
	LP	0.344	0.364	0.462	0.478	0.491
	Proposed	0.640	0.719	0.677	0.678	0.650
**Surveillance, Epidemiology, and End Results Colorectal Cancer**						
	GSSL	0.525	0.527	0.447	0.585	0.578
	LP	0.540	0.532	0.512	0.540	0.513
	Proposed	0.595	0.652	0.640	0.581	0.590
**Second Affiliated Hospital of Zhejiang University Colorectal Cancer**						
	GSSL	0.547	0.573	0.564	0.553	0.580
	LP	0.454	0.448	0.512	0.460	0.507
	GCN^d^	0.505	0.575	0.562	0.585	0.606
	Proposed	0.587	0.650	0.634	0.568	0.508

^a^AUC: area under the receiver operating characteristics curve.

^b^GSSL: graph-base semisupervised learning.

^c^LP: label propagation.

^d^GCN: graph convolutional network.

### Boosting Semisupervised Learning by Generating a Density Gap

In this section, we visualize the final layer of discriminator D in the proposed method by feeding it real samples from UCI-T2D and their generated counterparts. By embedding the output layer at different iteration steps with t-distributed stochastic neighbor embedding [22], the progression of the density gap from the generated samples, described in equation 4, is verified.

**Figure 3:**
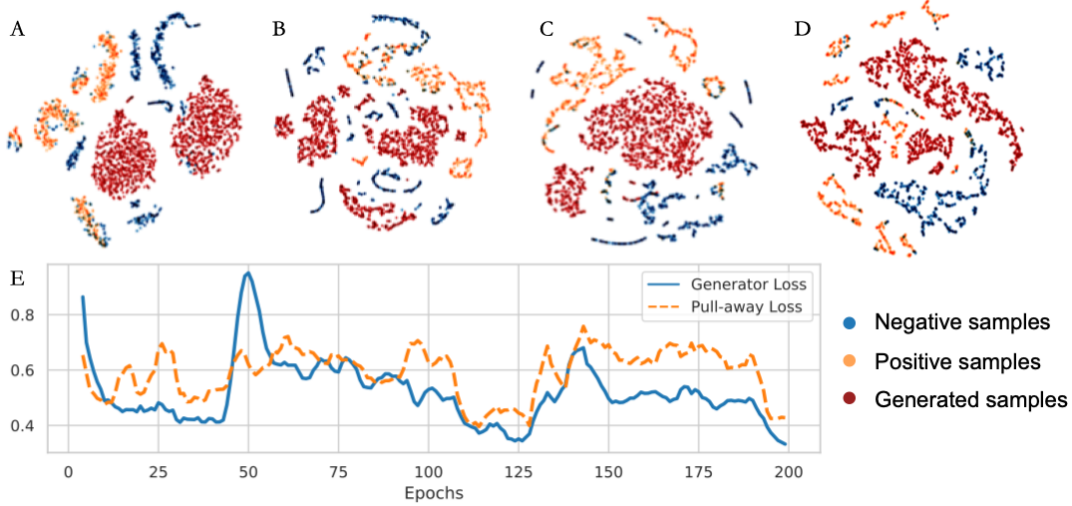
The progressive generation of density gaps in high-dimensional space and its visualization. (A) 0 epochs; (B) 40 epochs; (C) 80 epochs; (D) 120 epochs. The generated samples ultimately span the gap between a real and a false sample. The line chart (E) indicates the function of the pull-away term and how its optimization affects the generator and discriminator.

In Figure 3, we can see that at the starting epochs, the generated samples are mixed with the real samples, and the different classes are not divided. During training, D gradually learns a nonlinear map to project the fake samples and real samples into distinct clusters, while G learns to generate samples to take over the central area and isolate the clusters of different classes. This process has 2 advantages. First, the fake samples from the generator are unlikely to be copies of the original data, avoiding the direct disclosure of private information. Second, the samples on the borders of different classes are more correctly divided, which not only improves the accuracy of classification but also reveals the underlying training strategy of splitting one large class into several smaller classes to obtain a better classification.

### Fidelity Evaluation of the Generated Data

Frontier nodes are nodes at the borders of different clusters in a graph. The definition is given in Multimedia Appendix 1D. It is possible that a trained model is exploited directly for secondary purposes, such as fundamental profiling or developmental usage during the primary phase of data sharing [12]. We calculated the dimensionwise probability (DWPro) and dimensionwise prediction (DWPre) proposed by Choi et al [15] to evaluate the fidelity of the generator in our proposed model. DWPro is a basic statistical confirmation of the distributions of real data that are appropriately learned by the generator in the model. A training set R and synthetic sample set S of the same sample size are compared using the Bernoulli success probability pk of each dimension k. DWPre measures the extent to which the internal relations of every feature are captured. One dimension k is selected, and the rest of the features are used as training data. R and S are used to train the LR classifiers. Then, the dimension k is regarded as the label column for testing. It is a rational assumption that a smaller margin between the predictions of 2 models implies a better synthetic quality. The *F*_1_-score is selected as the metric for comparison.

**Figure 4:**
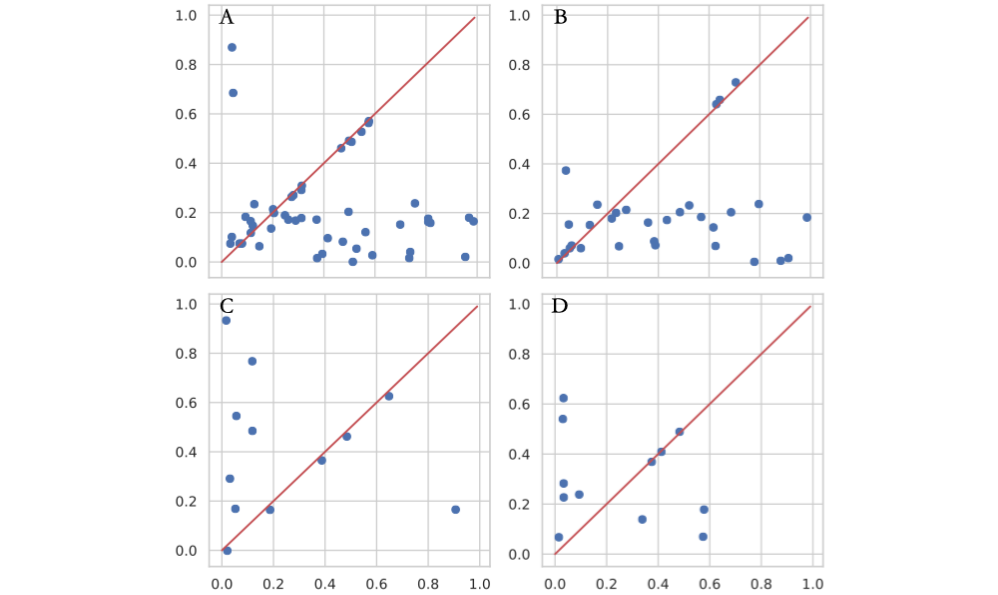
Dimensionwise probability of 4 selected data sets: (A) University of California Irvine Machine Learning Repository Type 2 Diabetes 30-Day Readmission; (B) Surveillance, Epidemiology, and End Results Ovarian Cancer; (C) Surveillance, Epidemiology, and End Results Colorectal Cancer; (D) Second Affiliated Hospital of Zhejiang University Colorectal Cancer. The x-axis is the Bernoulli success probability for the features of the real data sets, while the y-axis is the corresponding value from the synthetic data. Each blue dot represents a feature of the data set. The red diagonal line indicates an identical Bernoulli success probability of both the real and generated data sets, and ideal fidelity is learned featurewise by the generator.

**Figure 5:**
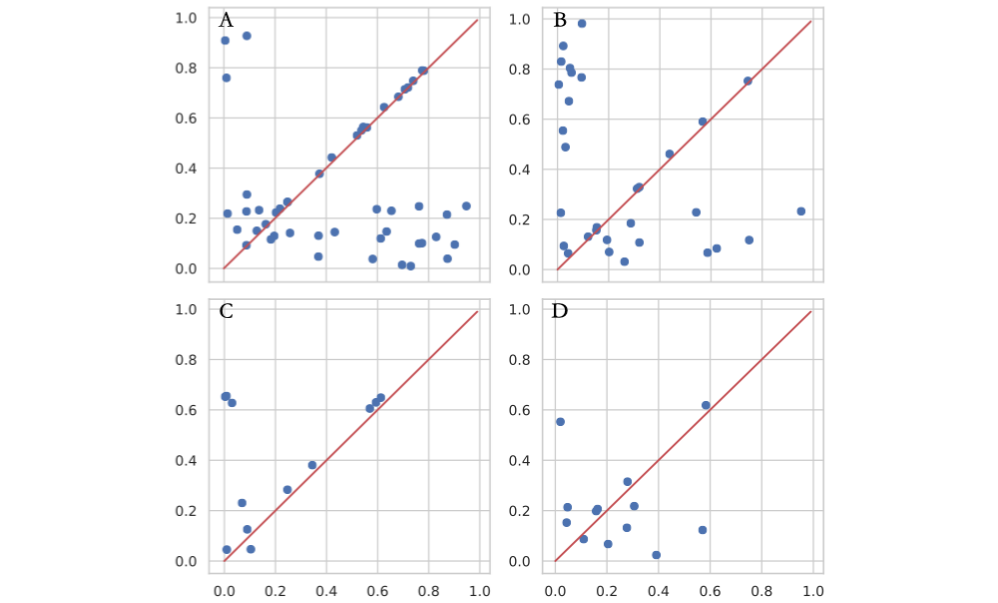
Dimensionwise prediction of 4 selected data sets: (A) University of California Irvine Machine Learning Repository Type 2 Diabetes 30-Day Readmission; (B) Surveillance, Epidemiology, and End Results Ovarian Cancer; (C) Surveillance, Epidemiology, and End Results Colorectal Cancer; (D) Second Affiliated Hospital of Zhejiang University Colorectal Cancer. The x-axis is the *F*_1_-score of models trained on the real data sets, while the y-axis is the corresponding values from the synthetic data. Each blue dot represents a feature of the data set. The red diagonal line indicates that the *F*_1_-score was identical for the models trained and tested on the real and generated data sets, and ideal interdimensional fidelity was learned by the generator.

Figure 4 shows that all 4 data sets were depicted well from a featurewise perspective, and over half of the dots are near the diagonal line. In Figure 4C, the consistency of each feature indicates high synthetic quality. Figure 5 shows a mildly diminished learning quality considering interdimensional fidelity. However, half of the features are still likely to be inferred from the remaining columns and the same proportion of features. Considering that the generated frontier is still different from the directly generated datapoints [15,16], the fidelity is acceptable for some secondary uses.

### Evaluation of the Disclosure Risk of the Generated Data

The generator in our proposed model may be exploited to generate data points similar to the original data sets, posing threats to patient privacy. We need to ensure that the frontier nodes generated by the proposed model can be protected from attackers with compromised data. Therefore, a quantitative evaluation of presence and attribute disclosure was conducted on the SAHZU-CRC data set. Of the real samples N, 1% were randomly sampled, and among the 11 dimensions (the numerical dimensions are left out and the nominal columns are collapsed into 2 for simplification), a progressively increasing number of features, denoted as r, were assumed to be known by the attacker. Then, the attacker could exploit the knowledge of the data (1% × N × r) to conduct k-NN searches of the synthetic data, and the other unknown feature values were estimated according to those of the k-NN. Finally, the unknown features were compared to the real features to gain precision and accuracy. The calculation was repeated 100 times with 1% of the real records chosen at random.

**Figure 6:**
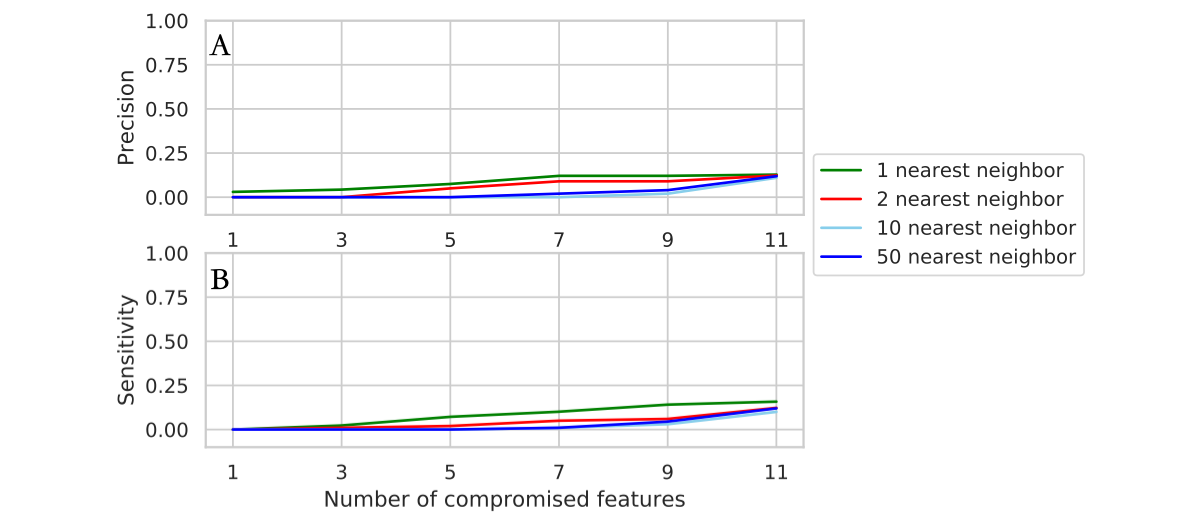
Privacy preservation evaluation. When increasing the number of known features, the attack achieves (A) precision and (B) sensitivity with 1% compromised records from the Second Affiliated Hospital of Zhejiang University data set.

Under these circumstances, the sensitivity indicates when the attacker has 1% × N × r of the disclosed data and all the synthetic data and how many records of all the positive features the attacker can correctly estimate with a 1-NN attack. The precision indicates how many features among all the features estimated positive by the attack were on average accurate. For instance, in Figure 6A, if an attacker with 1% of the records (12 of 1244 records for SAHZU-CRC) and 5 features from the real data conducts 1-NN on the synthetic SAHZU-CRC data generated by the proposed method, the positive estimation of the remaining unknown features of the real data will be 12.5% correct on average (0.125 precision), and of all the positive predictions, 15.8% will be correct (0.158 sensitivity).

In summary, the precision and sensitivity of attacks on synthetic data is relatively low, 0.158 at best when r is 11. The most effective attack setting is 1-NN. It is difficult to estimate more information from our frontier nodes due to the modification of the network losses. Substitution of both the generator and discriminator learning strategies boosts the model performance on classification with label deficiency and provides synthetic samples capable of preventing disclosure.

### Scalability of the Memory Consumption of Batch-Based Training

Because GPUs have been used in deep learning–based computation, we further examined whether the proposed method could achieve practical memory consumption. The aforementioned semisupervised methods are compared with our proposed method on memory consumption for 4 data sets. For the algorithms that do not need GPUs, their central processing unit consumption is measured.

For small data sets (eg, SAHZU-CRC), our proposed method takes up more space because of its complex network structure (Table 3). However, as the size expands, our proposed method shows the least and most stable space consumption, because minibatch training is independent of the number of samples (for SEER-CRC and SEER-OVC). Conventional network-based SSL methods tend to train on full batches. When the data set is large enough, there is a huge obstacle to storing the data in memory. Stable memory consumption implies a scalable model for training and prediction on diverse data sizes. The GCN, as a transductive SSL method, is unable to be directly scaled to larger data sets despite its excellent representative ability.

**Table 3:** Graphics processing unit memory consumption of the proposed method against that of typical semisupervised learning algorithms on 4 data sets. The graph convolutional network is not suitable for data sets other than Second Affiliated Hospital of Zhejiang University; therefore, only one result is shown.

	UCI-T2D^a^, MB	SEER-OVC^b^, MB	SEER-CRC^c^, MB	SAHZU-CRC^d^, MB
Graph-based semisupervised learning (CPU^e^)	1374	770	1260	297
Label propagation (CPU)	1200	702	1263	257
Graph convolutional network	Out of memory	Out of memory	Out of memory	732
Proposed	336	345	330	332

^a^UCI-T2D: University of California Irvine Machine Learning Repository Type 2 Diabetes 30-Day Readmission.

^b^SEER-OVC: Surveillance, Epidemiology, and End Results–Ovarian Cancer.

^c^SEER-CRC: Surveillance, Epidemiology, and End Results–Colorectal Cancer.

^d^SAHZU-CRC: Second Affiliated Hospital of Zhejiang University.

^e^CPU: central processing unit.

## Discussion

### Principal Results

The proposed model fully utilizes the inner graphical structure of EHRs and provides cost-effective prediction metrics. The density gap derived from the modified network loss enables different class labels to be better distinguished. Under label-deficient circumstances, the proposed model achieves a comparable performance to that of conventional supervised learning methods where all of the training data are labeled. Specifically, with only 10% labeled data, the performance of popular supervised machine learning methods is approached, which implies there is a broad set of situations in which this model could be considered for prediction tasks. Following the same setting of label rates for the purpose of comparison, the conventional SSL methods show poor data representation ability. The learning performance, compared to that of our proposed method, shows worse stability and scalability. With the increasing label rate, the conventional SSL models display either poor performance on classification due to label deficiency or extreme cases where the classifier puts every sample into 1 class as a consequence of overfitting. Additionally, the memory cost is worth noting. Most semisupervised methods have a tendency to copy the whole graph structure into memory [19,27,43], which brings a very large burden of computational resources considering that the EHRs absorb increasingly more data.

Extracting the frontier of generated samples that shows high performance in DWPro and DWPre has potential in applying some special frontier nodes as sample data for secondary usage, in the same way as related work applies GANs to RS-EHR generation. According to related studies [15,16], generating data with adequate quality is crucial in cross-organization data sharing. The quality of the data determines the model performance on realistic data sets. Additionally, for diverse developmental needs, the more realistic the generated data are compared to the real samples, the more persuasiveness and fidelity the researching systems will acquire. The generator in our model fulfills this demand by generating similar samples to the original data after the training phase.

To reveal the hidden clinical and physiological characteristics of certain groups, EHRs are among the most reliable information sources. Nonetheless, administrative regulations and the protection of patient privacy have decreased the accessibility of EHRs for a variety of reasons and made downstream analysis inconvenient. Our method first addresses privacy considerations by transforming the data set into a k-NN graph where the similarities between different patients are re-encoded while the identifying information is hidden. Second, the vector from the embedded graph is fed into our model for further analysis. Under practical scenarios, authorization to share and use the original data will not be a necessity. Additionally, even when conventional attacks attempt to reidentify personal information from the publicly generated samples, the k-precision and k-sensitivity metrics indicate that it is quite safe if the attacker holds only a small fraction of the knowledge of the real data and conducts the most powerful 1-NN attack. Furthermore, the density gaps avoid the usual case where GANs would otherwise be trained to copy the real input, thereby shielding the patient information from another possible method of disclosure.

### Limitations

The limitations of this study are still worth noting. The evaluation of how the proposed model can improve data quality and predict performance on the actual label collection phase has yet to be considered. Additionally, we excluded all patient duplicates to conduct a prediction method without considering any temporal information. Further investigation of the temporal trajectories of the same patients may reveal more of the inner mechanisms of disease progression, and localization methods of temporal and spatial structure in many other fields may address the same problem [44,45]. Additionally, the proposed model only applies to some label-rate setups, and performance diminishes as more labels become available. The thresholds for switching between the different algorithms (SSL and SL) remain to be studied. Finally, to be more protective of patient privacy and intellectual property, our future explorations include graph generation and attention mechanisms [28,29,34,46]. A whole generated graph can be taken into consideration. With the power of GANs, the underlying structure of large-scale EHRs could be preserved while achieving full anonymity.

### Conclusions

EHR-based systems and observational studies with conventional learning strategies are facing diverse challenges as data and label inaccessibility increase. Training on few labeled data is a pivotal task and needs substantial resources. Uncovering the underlying graphical structure of EHRs brings a motivating perspective and informative prerequisites to analyzing patient data. As a downstream analysis method, GAN-boosted SSL uses a graphical structure and greatly improves learning quality in label-deficient situations. GANs with refined loss also meet the demands of deidentification and decent data fidelity under multiple-source data-sharing circumstances. This combination achieves impressive performance on prediction metrics, data quality, and protection from compromising attackers over various data sets, while popular machine learning methods encounter obstacles to sufficient training. This study indicates the potential of discovering the structural features that underlie the data instead of merely feeding models coordinated data sets and using unlabeled data when label deficiency occurs.

## Appendix

Multimedia Appendix 1 Graph structure definition and semisupervised learning on graph formularization.

## Abbreviations

AUC: area under the receiver operating characteristics curve
DWPre: dimensionwise precision
DWPro: dimensionwise probability
EHR: electronic health record
eMERGE: Electronic Medical Records and Genomics
GAN: generative adversarial network
GCN: graph convolutional network
GPU: graphics processing unit
GSSL: graph-base semisupervised learning
GSSL: graph-based semisupervised learning
k-NN: k-nearest neighbors
LP: label propagation
LR: logistic regression
RF: random forest
RS-EHR: realistic synthesized electronic health record
SAHZU-CRC: Second Affiliated Hospital of Zhejiang University Colorectal Cancer
SEER-CRC: Surveillance, Epidemiology, and End Results Colorectal Cancer
SEER-OVC: Surveillance, Epidemiology, and End Results Ovarian Cancer
SL: supervised learning
SSL: semisupervised learning
SVM: support vector machine
UCI-T2D: University of California Irvine Machine Learning Repository Type 2 Diabetes 30-Day Readmission
