# Supplementary material for: Improving an Electronic Health Record–Based Clinical Prediction Model Under Label Deficiency: Network-Based Generative Adversarial Semisupervised Approach
Source: JMIR Med Inform. 2023 Jun 13;11:e47862. doi: 10.2196/47862 (PMC10337516; doi:10.2196/47862)
Supplement: Multimedia Appendix 1 [file medinform_v11i1e47862_app1.docx]

# Appendix A. Graph Structure and Semisupervised Learning on Graphs

Raw data can be denoted as $X=\{x_{1},x_{2},\ldots,x_{N}{\}}^{\top}$ , where the column vector $x_{i}\in R^{d}$, *d* is the dimension of the data points, and $N = |X|$ is the number of data points. Denote $G = (V, E)$ as a graph, where $V$ is its node set and $E \subseteq V \times V$ is its edge set. A node element of $E$ is denoted as $v_{j}$ , and an edge element of $E$ connecting node $v_{i}$ and node $v_{j}$ is denoted as $e_{ij}$; its value, denoted as $w_{ij}$ , is either binary or weighted. The adjacency matrix of $G$ is denoted as $A$. Each node $v_{i}$ has its own feature vector $x_{i}\in R^{d}$ with dimension *d* and a label $\mathrm{yi}\in\{0, 1, \cdot\cdot\cdot, M - 1\}$, where M is the number of classes. Let the set of labeled nodes be denoted as $V^{L}$, and the set of unlabeled nodes as $V^{U}$ . Under SSL circumstances, only a small subset of nodes are labeled. Thus, $V=V^{L}\cup V^{U}, V^{L} \cap V^{U} = \emptyset and |V^{L}| \ll|V^{U}|$ .

Then, we define the semisupervised problem on this graph. Given a partially labeled graph $G = (V^{L}\cup V^{U} , E)$, the objective is to learn a function *f* using the node features and graph structure to predict the labels of the unlabeled nodes. This schedule is called transductive learning, in which training and prediction are performed simultaneously on a given database. The SSL on the graph can be formalized as:

$f^{*} = argmin||Y^{U} - f(X^{U};A,V^{L})||$*,* (A.1)

*f*

where $X^{U}=\left( x_{1},x_{2},\ldots,x_{\left| V^{U} \right|} \right)^{\top}$, $Y^{U}=\left( y_{1},y_{2},\ldots,y_{\left| V^{U} \right|} \right)^{\top}$,, and $||\cdot||$is the loss function between the predicted and true labels.

# Appendix B. Data Preprocessing

The categorical features were filtered and mapped following a pipeline: (1) drop the variables that have the same values in all the records or are missing in over 98% of the records; (2) create new features by merging features that have the same implications according to common knowledge; (3) re-encode medications from text descriptions to categorical variables; (4) collapse the coding features to smaller label sets; (5) remove records with duplicated patient identifiers; and (6) one-hot encode categorical features to be part of the final feature vectors. The numerical features were processed as follows: (1) transformation by inverse hyperbolic sine with a coefficient of 5 if the kurtosis was over a threshold; (2) rescaling of each column value by its 99.5th percentile overall. A brief characteristic summary of the selected datasets is given below.

## Appendix B.1. Data Description

Table B.1: Demographic and clinical characteristics of UCI-T2D patients (n=61675). Twenty-three drug usage columns, 11 cross-term columns, and 8 diagnosing code columns are left out.

| Variable | count or mean | percentage(%) or std |
| --- | --- | --- |
| Race | | |
| Caucasian | 46117 | 74.77 |
| Hispanic | 1319 | 2.14 |
| Asian | 430 | 0.70 |
| Other | 2697 | 4.37 |
| Gender | | |
| Male | 32811 | 53.20 |
| Female | 28864 | 46.80 |
| Age (years) | | |
| [70-80) | 15663 | 25.40 |
| [60-70) | 13804 | 22.38 |
| [50-60) | 10829 | 17.56 |
| [80-90) | 9867 | 16.00 |
| [40-50) | 5989 | 9.71 |
| [30-40) | 2357 | 3.82 |
| [90-100) | 1583 | 2.57 |
| [20-30) | 982 | 1.59 |
| [10-20) | 468 | 0.76 |
| [0-10) | 134 | 0.22 |
| Time in hospital (days) | 4.28 | 2.94 |
| Number of lab procedures | 42.92 | 19.90 |
| Number of procedures | 1.42 | 1.75 |
| Number of medications | 15.67 | 8.28 |
| Number of outpatients | 0.28 | 1.06 |
| Number of emergencies | 0.10 | 0.51 |
| Number of inpatients | 0.18 | 0.60 |
| Number of diagnoses | 7.23 | 2.00 |
| Max glucose in serum (mg/dL) | | |
| None | 58711 | 95.19 |
| Norm | 1503 | 2.44 |
| >200 | 828 | 1.34 |
| >300 | 632 | 1.03 |
| Hemoglobin A1C (%) | | |
| None | 50399 | 81.72 |
| >8 | 5471 | 8.87 |
| Norm | 3290 | 5.33 |
| >7 | 2515 | 4.08 |
| Admission type ID | | |
| Elective | 12145 | 0.20 |
| Not available | 6647 | 0.11 |
| Discharge disposition ID | | |
| To another hospital | 11806 | 0.19 |
| Left AMA | 351 | 0.01 |
| Neonate discharged to another | 49 | 0.00 |
| hospital for neonatal aftercare | | |
| Not available | 2960 | 0.05 |
| Admission source ID | | |
| Transferred from a hospital | 4307 | 0.07 |
| Emergency room | 28754 | 0.47 |
| Not available | 4271 | 0.07 |
| Readmitted | | |
| NO | 36689 | 59.49 |
| >30 | 19474 | 31.57 |
| <30 | 5512 | 8.94 |

Table B.2: Demographic and clinical characteristics of SEER-OVC patients (n=10038).

| Variable | count or mean | percentage(%) or std |
| --- | --- | --- |
| Race | | |
| white | 8288 | 82.57 |
| Other | 1146 | 11.42 |
| Black | 604 | 6.02 |
| Primary Site | | |
| C56.9 | 9230 | 91.95 |
| C57.0 | 806 | 8.03 |
| Other | 2 | 0.02 |
| Grade | | |
| III | 4190 | 41.74 |
| IV | 3152 | 31.40 |
| II | 1693 | 16.87 |
| I | 1003 | 9.99 |
| Laterality | | |
| Bilateral, single primary | 3738 | 37.24 |
| Right - origin of primary | 3152 | 31.40 |
| Left - origin of primary | 3061 | 30.49 |
| Paired site | 87 | 0.87 |
| Surgery primary site code | | |
| 0 | 9909 | 98.71 |
| 20-90 | 129 | 1.29 |
| CS extension code | | |
| [700,800] | 5255 | 52.35 |
| [100,500) | 3250 | 32.38 |
| [500,700) | 1521 | 15.15 |
| 950 | 10 | 0.10 |
| Other | 2 | 0.02 |
| CS lymph node code | | |
| III | 7706 | 76.77 |
| I | 2332 | 23.21 |
| IV | 1539 | 15.33 |
| II | 1183 | 11.79 |
| Derived AJCC T, 6th ed | | |
| T3 | 5296 | 52.76 |
| T1 | 3179 | 31.67 |
| T2 | 1553 | 15.47 |
| T0,TX | 10 | 0.10 |
| Derived AJCC N, 6th ed | | |
| N0,NX | 7702 | 76.73 |
| N1 | 2336 | 23.27 |
| Derived AJCC M, 6th ed | | |
| M0 | 8499 | 84.67 |
| M1 | 1539 | 15.33 |
| CS mets at dx code | | |
| 0 | 8498 | 84.66 |
| 10-70 | 1540 | 15.33 |
| Sequence number | | |
| One primary only | 7538 | 75.09 |
| More primaries | 2500 | 24.91 |
| First malignant primary indicator | | |
| Yes | 8553 | 85.21 |
| No | 1485 | 14.79 |
| Scope Reg LN Sur | | |
| regional lymphs | 6513 | 64.88 |
| None | 3466 | 34.53 |
| Sentinel node biopsy | 59 | 0.59 |
| Histologic type ICD-O-3 | | |
| 8260,8590,8631,8634,8640,8670 | 203 | 2.02 |
| 9060,9071,9080,9085,9090,8041 | 74 | 0.74 |
| 8890 | 2 | 0.02 |
| Other | 9759 | 97.22 |
| CS tumor size (mm) | 103.33 | 112.75 |
| Regional nodes examined | 10.65 | 15.27 |
| Regional nodes positive | 34.97 | 46.07 |
| Age (years) | | |
| [55,59] | 1487 | 0.15 |
| [60,64] | 1464 | 0.15 |
| [50,54] | 1390 | 0.14 |
| [65,69] | 1221 | 0.12 |
| [45,49] | 982 | 0.10 |
| [70,74] | 902 | 0.09 |
| [75,79] | 690 | 0.07 |
| [40,44] | 568 | 0.06 |
| [80,84] | 435 | 0.04 |
| [35,39] | 249 | 0.02 |
| >=85 | 237 | 0.02 |
| [30,34] | 142 | 0.01 |
| [25,29] | 101 | 0.01 |
| [20,24] | 86 | 0.01 |
| [15,19] | 54 | 0.01 |
| [10,14] | 26 | 0.00 |
| [5,9] | 3 | 0.00 |
| [1,4] | 1 | 0.00 |
| Survival months | 58.50 | 58.50 |
| Vital status recode | | |
| Alive | 5247 | 52.27 |
| Dead | 4791 | 47.73 |

Table B.3: Demographic and clinical characteristics of SEER-CRC patients (n=40014).

| Variable | count or mean | percentage(%) or std |
| --- | --- | --- |
| CS tumor size | 45.52 | 30.14 |
| Lymph node positive | 1.48 | 3.19 |
| Age |  |  |
| Other | 10381 | 25.67 |
| <75 | 10402 | 25.72 |
| <65 | 9867 | 24.40 |
| <55 | 9364 | 23.15 |
| Gender |  |  |
| Male | 20342 | 50.30 |
| Female | 19672 | 48.64 |
| Grade |  |  |
| Moderately differentiated | 29464 | 72.86 |
| Well differentiated | 6491 | 16.05 |
| Poorly or none | 4059 | 10.04 |
| Histology |  |  |
| Adenocarcinoma | 30042 | 74.29 |
| Other | 6028 | 14.91 |
| Mucinous OR Signet ring cell | 3944 | 9.75 |
| Site |  |  |
| Colon | 30757 | 76.05 |
| Rectum | 9257 | 22.89 |
| AJCC T stage |  |  |
| T3 | 23733 | 58.69 |
| T2 | 8590 | 21.24 |
| T1 | 4727 | 11.69 |
| T4a | 1774 | 4.39 |
| T4b | 1190 | 2.94 |
| AJCC N stage |  |  |
| N0 | 26895 | 66.50 |
| N1a | 4723 | 11.68 |
| N1b/N1c | 4345 | 10.74 |
| N2a | 2511 | 6.21 |
| N2b | 1540 | 3.81 |
| Vital status |  |  |
| Alive | 34978 | 86.49 |
| Dead | 5036 | 12.45 |

Table B.4: Demographic and clinical characteristics of SAHZU-CRC patients (n=1244).

| Variable | count or mean | percentage (%) or std |
| --- | --- | --- |
| Age(years) | 58.9 | 12.41 |
| Histology |  |  |
| Adenocarcinoma | 1116 | 89.71 |
| Mucinous OR Signet ring cell | 126 | 10.13 |
| Other | 2 | 0.16 |
| Tumor grade |  |  |
| Well differentiated | 464 | 37.30 |
| Moderately differentiated | 224 | 18.01 |
| Poorly or none | 207 | 16.64 |
| Not available | 349 | 28.05 |
| AJCC T 7th edition |  |  |
| T2 | 699 | 56.19 |
| T4a | 449 | 36.09 |
| T1 | 77 | 6.19 |
| T4b | 19 | 1.53 |
| AJCC N 7th edition |  |  |
| N0 | 797 | 64.07 |
| N1b/N1c | 174 | 13.99 |
| N1a | 142 | 11.41 |
| N2a | 80 | 6.43 |
| N2b | 51 | 4.10 |
| AJCC stage 7th edition |  |  |
| II | 510 | 41.00 |
| IIIb | 302 | 24.28 |
| I | 288 | 23.15 |
| IIIc | 92 | 7.40 |
| IIIa | 52 | 4.18 |
| Carcinoembryonic antigen (CEA) | 10.84 | 0.87 |
| levels (ng/ml) |  |  |
| Gender |  |  |
| Male | 744 | 59.81 |
| Female | 500 | 40.19 |
| Site |  |  |
| Colon | 673 | 54.10 |
| Rectum | 571 | 45.90 |
| Neural invasion status |  |  |
| No invasion | 1207 | 97.03 |
| Invasion | 37 | 2.97 |
| Survival |  |  |
| Alive | 1033 | 83.04 |
| Dead | 211 | 16.96 |

# Appendix C. Basic GAN Loss and its Optimization

In general, a GAN is composed of two networks, a generator *G* and a discriminator *D*. The network is trained by estimating a generative model in an adversarial manner, whereby *G* is trained to approximate the distribution of the real data and *D* is trained to distinguish the real samples from the generated samples. The whole process can be formalized as a min-max loss: $\min_{G}{max}_{D}V\left( G,D \right)=E_{x\sim P_{d}\left( x \right)\mathbb{log}} \left[ D\left( x \right) \right]+E_{z\sim P_{z}\left( z \right)\mathbb{log}} \left[ \mathbb{1-}D\left( G\left( z \right) \right) \right]$*,* (C.1)where P*d* is the data distribution from the training data and $Pz(z)$ is a prior on the input noise. The state-of-the-art well-accepted loss for basic GAN training [32, 33] is illustrated in the appendix, and the respective loss for the generator and discriminator is denoted as ${loss}_{Gwgp}$ and ${loss}_{Dwgp}$. We treat these as basic terms in both the generator and discriminator training loss.

Generative models for graphs can be classified by their objectives. (1) Network generation [29, 30]: The global topological structure is learned as a set of traverses and embeddings from existing real graphs, but the methods do not apply to massive databases due to memory limitations. (2) Edge generation [34]: The generator is trained to maximize the possibility that a generated edge exists, which is determined by the node features of the two nodes at its ends. (3) Node feature generation [31]: This strategy is the most intuitive for most converted coordinated datasets and has excellent scalability for its mini-batch training process.

The losses of GANs are crucial in training. The quality, diversity and stability of the generated samples are determined by the loss. The stateof-the-art training strategy is the Wasserstein GAN with gradient penalty (WGAN-GP), in which the distance between $P_{d}(x)$ and $P_{G(z)}(x)$is continuously quantified by the Wasserstein distance and stable optimization is enabled through the gradient-penalized term. The general objective can be described as:

$\min_{D}{loss}_{Dwgp}= -D(G(z))$ (C.2)

$\min_{G}loss_{Gwgp}=D\left( G\left( z \right) \right)-D\left( x \right)+\lambda\left( \left| \left| \partial_{\hat{x}}D\left( \hat{x} \right) \right| \right|_{2}-1 \right)^{2}$ (C.3)

Where $\hat{x} = \alpha x + \left( 1-\alpha\right) G\left( z \right)$ indicates interpolation between the real samples and generated samples.

The refining of loss can serve not only to approximate the original distribution but also to boost SSL. The output layer of *D* is set to $y \in R^{M+1}$, where the dimension includes the probabilities of the input being a real sample that belongs to a certain class $y^{\left( M \right)}=0,\sum_{i=0,\cdot\cdot\cdot,M-1} y^{\left( i \right)}=1$ and of being a fake sample *y*^(^*^M^*^)^ = 1.

# Appendix D. Frontier Nodes

We define frontier nodes, as in Figure D.1, in a graph as nodes whose neighbors include nodes of the same classes and different classes. These nodes are essential in SSL. To achieve perfect classification, the frontier nodes need to be classified correctly. The generated samples should be connected to frontier nodes as their 1-hop neighborhoods. Considering privacy preservation, frontier nodes are also crucial in this problem. Most generated samples are not similar to real samples in the embedded space and are therefore not considered generated samples. However, frontier nodes are actually quite close to generated samples and therefore might be exploited to reidentify real data. If each feature of the generated samples shows sufficient fidelity under examination, we can ensure that the generator is capable of certain secondary usage cases. For the original dataset *R*, the synthetic sample set *S*, and their network *G*, we denote the frontier nodes as *F*:

$F = \{f|N(f) \cap R \neq\emptyset,N(f) \cap S 6\neq\emptyset,f \in S\}$*.* (D.1)


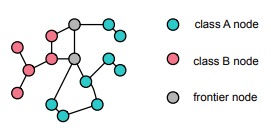


Figure D.1: Definition of the 1-hop frontier.
